# Correlation Between High Serum Ferritin Level and Gestational Diabetes: A Systematic Review

**DOI:** 10.7759/cureus.18990

**Published:** 2021-10-23

**Authors:** Lubna Durrani, Saman Ejaz, Lorena B Tavares, Moiud Mohyeldin, Deya Abureesh, Mustafa Boorenie, Safeera Khan

**Affiliations:** 1 Obstetrics and Gynecology, California Institute of Behavioral Neurosciences & Psychology, Fairfield, USA; 2 Bioethics, Columbia University, New York, USA; 3 Internal Medicine/Research, California Institute of Behavioral Neurosciences & Psychology, Fairfield, USA; 4 Research, California Institute of Behavioral Neurosciences & Psychology, Fairfield, USA; 5 Neurosurgery/Research, California Institute of Behavioral Neurosciences & Psychology, Fairfield, USA; 6 Internal Medicine, California Institute of Behavioral Neurosciences & Psychology, Fairfield, USA

**Keywords:** hepcidin, stfr, pancreatic beta-cell, insulin resistance, pregnancy, gestational diabetes, hemoglobin, ferritin

## Abstract

Gestational diabetes mellitus (GDM) is a growing pregnancy-related health problem all over the world. It has been noticed that women with high serum ferritin levels have a strong relationship with GDM by increased insulin resistance and increased insulin secretion from the pancreas resulting in pancreatic beta-cell exhaustion. Heme iron is also responsible for increasing the body's iron store and hence causing oxidative injury to pancreatic cells.

In this systematic review, we researched the association between high serum ferritin levels and GDM. Three databases were consulted for articles related to GDM and high ferritin. These include Medical Literature Analysis and Retrieval System Online (MEDLINE), PubMed, and PubMed Central (PMC). Additional articles were retrieved from the institutional database. After filtering, 10 articles were finally selected, and quality was checked using the Joanna Briggs Institute (JBI) Critical Appraisal quality check tool. Serum iron biomarkers including ferritin, iron, and soluble transferrin receptor (sTfR) were measured.

Our systematic review indicates that high maternal serum ferritin has a significant role in the development of GDM. We have also noticed the importance of sTfR and serum hepcidin as biomarkers to monitor high ferritin levels. Our study also observed a positive relationship between high heme iron intake and gestational diabetes mellitus. Therefore, more research is required to understand this relationship to identify populations at risk.

## Introduction and background

According to the World Health Organization (WHO), anemia affects 40% of pregnant women worldwide and continues to be a global health crisis wherein iron deficiency anemia is more prevalent. Iron is a significant component of human cells and a major component of red blood cells. Iron deficiency anemia can alter the feto-maternal iron homeostasis [1-3]. This results in increased maternal and fetal complications, including premature delivery, intrauterine growth restriction, and neonatal and perinatal death [4]. The WHO recommended dietary intake for pregnant women is 30-60 mg of elemental iron to prevent iron deficiency anemia in mothers [5]. The health policy for iron supplementation differs between countries. Nonetheless, an iron overload can develop due to an excessive accumulation of this mineral and has been implicated in the development of various illnesses such as diabetes mellitus and heart failure [6-8].

Studies on the general population have determined an association between high dietary intakes of iron through animal sources or supplementation and risk of type 2 diabetes mellitus (T2DM) [9-10]. Moreover, moderately elevated ferritin levels are linked with increased insulin resistance, reduced insulin secretion, and T2DM [9-11]. The relationship between excessive iron and T2DM has raised concerns regarding their impact on gestational diabetes mellitus (GDM). Iron intake, serum iron, and serum ferritin can aid in assessing iron levels in the human body. These measurements can be employed to diagnose normal iron, iron deficiency anemia, or iron overload [12]. Even though serum ferritin does not directly evaluate amounts of circulating iron, it can help recognize iron overload [12-13].

Any degree of hyperglycemia that is identified at the onset of pregnancy or diagnosed for the first time during pregnancy is defined as GDM [14]. Of all pregnancies, 0.5-15% are affected by GDM, leading it to become a worldwide pregnancy-associated health issue [15]. GDM is positively associated with adverse perinatal outcomes including neonatal hypoglycemia, jaundice, and perinatal mortality; furthermore, maternal complications associated with GDM are pregnancy-induced hypertension, shoulder dystocia, and increased risk of cesarean deliveries. GDM also increases the risk of maternal and newborn cardiovascular disease, obesity, and the risk of development of diabetes mellitus later in life [16,17].

In the human body, iron is stored in the form of ferritin and hemosiderin [13]. Patients with GDM were discovered to have higher iron stores, which are measured most commonly by serum ferritin. Moreover, elevated levels of hemoglobin in early pregnancy have been identified as an independent GDM risk factor [18-19]. Furthermore, lower hemoglobin levels and anemia during pregnancy have been associated with a lower risk of GDM [20].

Although numerous studies have been conducted, these studies produce inconsistent findings of the relationship between high serum ferritin and GDM. Further studies are warranted to better characterize iron's role in the pathophysiological pathways related to GDM and identify high-risk populations.

This systematic review provides an overview of the studies done so far to find the link between high serum ferritin level and developing GDM. This will facilitate the identification of pregnancies wherein there is a risk of developing GDM.

## Review

Methods

**Figure 1:**
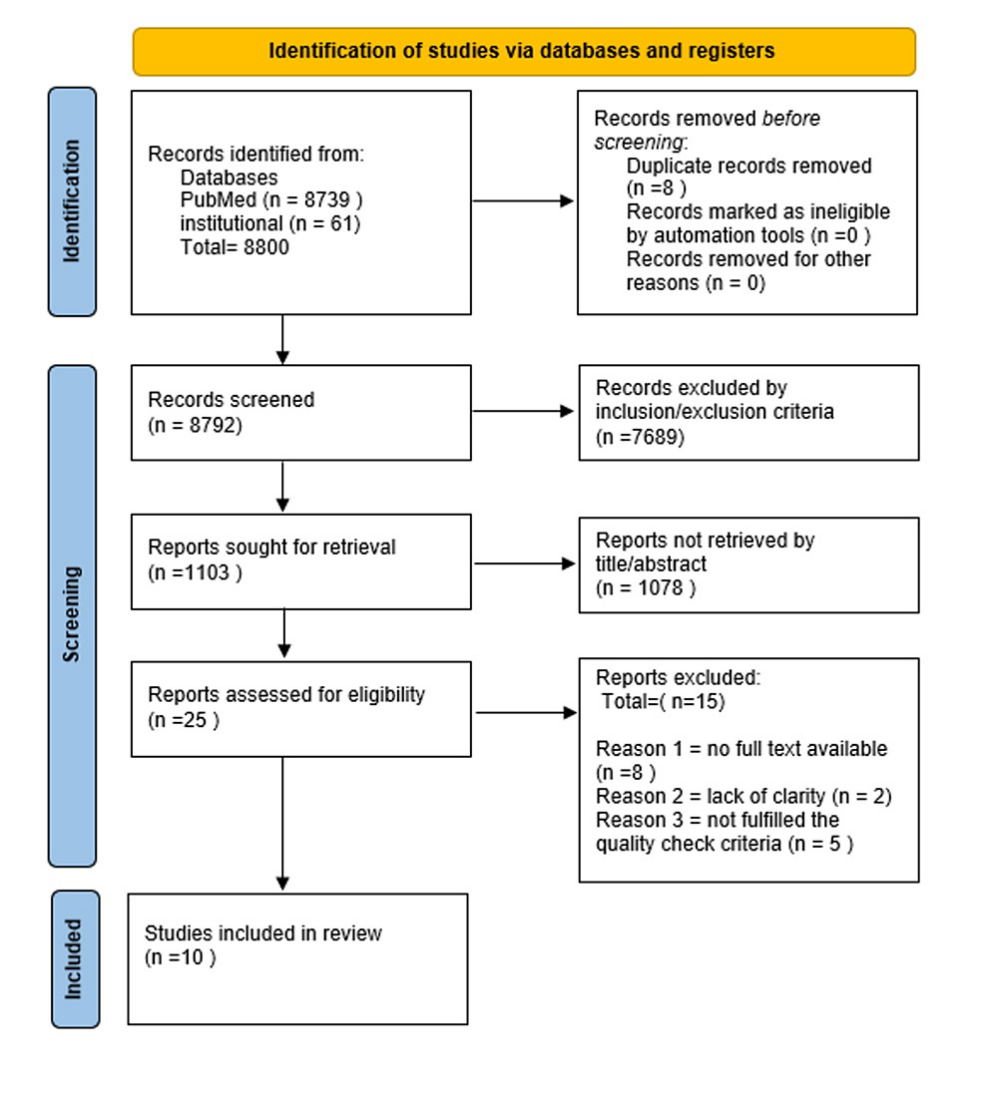
Selection of studies as illustrated by the PRISMA flow chart. PRISMA, Preferred Reporting Items for Systematic Reviews and Meta-Analyses.

Our systematic review follows Preferred Reporting Items for Systematic Reviews and Meta-Analyses (PRISMA) 2020 (Figure 1) [21].

Inclusion and Exclusion Criteria

Human studies of the past 10 years pertaining to pregnancies at any gestational age written in English were retrieved. Additionally, case-control studies and cohort studies describing the association of GDM with iron biomarkers were included. Pregnancies with a history of type 1 or type 2 diabetes mellitus or any inflammatory or infectious disease were excluded. All animal studies were removed. Furthermore, grey literature was not included.

Search Strategy

Studies from the past 10 years were selected from PubMed, Medical Literature Analysis and Retrieval System Online (MEDLINE), PubMed Central (PMC), and the institutional database. Studies are systematically searched by using keywords specific to the research. The search strategy includes keywords in Medical Subject Heading (MeSH) and Boolean operators "OR" and "AND." Keywords used were ferritin, hemoglobin, GDM, and pregnancy.

MeSH strategy was as follows: ferritin OR hemoglobin OR iron OR hemoconcentration ("Ferritins/adverse effects"[Majr] OR "Ferritins/blood"[Majr] OR "Ferritins/metabolism"[Majr] ) AND pregnancy OR gestation ("Pregnancy/complications"[Mesh] OR "Pregnancy/drug effects"[Mesh] OR "Pregnancy/metabolism"[Mesh] ) AND GDM OR gestational diabetes OR gestational diabetes mellitus OR diabetes mellitus OR HbA1c OR hyperglycemia OR glucose intolerance ("Diabetes, Gestational/diagnosis"[Mesh] OR "Diabetes, Gestational/etiology"[Mesh] OR "Diabetes, Gestational/metabolism"[Mesh] OR "Diabetes, Gestational/pathology"[Mesh]).

Data Gathering and the Search Procedure

As previously mentioned, this systematic review followed the reporting guidelines outlined by PRISMA 2020.

By searching four databases, PubMed, Medline, PMC, and the institutional database, 8,800 total records were identified. After removing eight duplicates, 8,792 records were screened based on their titles, abstracts, and inclusion and exclusion criteria. Thirty records were assessed based on the eligibility criteria, and the irrelevant studies were removed, while the remaining studies were read in the full text before selection. Finally, 10 articles were selected for review. Three investigators (LD, SE, and MM) independently reviewed the studies in full text, and no automation was used. Data were collected and tabulated with the author's name, year, location, study design, sample size, iron indices used for blood tests, and gestational age.

Quality Assessment and Risk of Bias

All the selected studies are observational studies that were quality assessed based on the Joanna Briggs Institute (JBI) Critical Appraisal tool. The selection criteria were based on 12 items. As high-quality studies were selected, the risk of bias was low.

Results

After searching different databases, a total of 8,800 studies were identified. Out of these, only 10 fulfilled the criteria and were finally selected for review. All the selected studies were observational studies; six were prospective cohorts and four were case-control studies. The population researched includes Iran, India, the USA, Denmark, Australia, Lebanon, and Finland, showing marked diversity in relation to ethnicity.

The total number of subjects was 8,070. In six studies, initial iron blood indices were conducted in the first trimester, and in the remaining four, this procedure occurred in the second trimester. Different criteria made the diagnosis of GDM. WHO criteria had been used in four studies, while another four studies employed the Carpenter-Coustan criteria. One study used the American Diabetic Association criteria, and in one study, the method of diagnosis was not mentioned. Out of these 10 studies, five were from Asia, one each from Australia, the USA, and the Middle East, and two from North Europe. Six studies selected in this systematic review were prospective cohort studies, they were by Soheilykhah et al. (2017), Bowers et al. (2016), Khambalia et al. (2016), Zein et al. (2015), Behboudi-Gandevani et al. (2013), and Helin et al. (2012), while four studies were case-control studies by Yadav et al. (2017), Rawal et al. (2017), Amiri et al. (2013), and Javadian et al. (2014) (Table 1).

**Table 1:** Characteristics of studies included in this systematic review. ADA = American Diabetes Association; GDM = gestational diabetes mellitus; BMI = body mass index; CRP = C‐reactive protein; WHO = World Health Organization; IADPSG = International Association of Diabetes and Pregnancy Study Groups; sTfR = serum transferrin receptor; TIBC = total iron-binding capacity; PIH = pregnancy-induced hypertension; LDL = low-density lipoprotein.

Authors	Year	Location	Study design	Sample size	Diagnosis of GDM	Trimester for biomarker collection	Iron indices	Variables adjustment
Yadav et al. [22]	2017	India	Case-control	30/60	Carpenter and Coustan criteria	Second	Serum ferritin	Obesity and history of infection
Soheilykhah et al. [23]	2017	Iran	Prospective cohort	1279	WHO	First	Serum ferritin, iron, hemoglobin, TIBC	Age, level of education, reproductive medical history
Bowers et al. [24]	2016	Denmark	Prospective cohort	350/349	WHO	First	Serum ferritin, sTfR, heme and non-heme dietary iron, iron supplement	Family history of diabetes, BMI, age, parity, CRP, oxidized LDL
Rawal et al. [25]	2017	USA	Case-control	107/321	WHO	First	Serum ferritin, sTfR, CRP	Age ethnicity, parity, education, smoking, alcohol, BMI, family history of diabetes
Khambalia et al. [26]	2015	Australia	Prospective cohort	3776	Unexplained	First	Serum ferritin, sTfR, CRP	Age, country of birth, BMI, smoking, PIH
Zein et al. [27]	2015	Lebanon	Prospective cohort	16/88	WHO and IADPSG	First	Serum ferritin, hemoglobin CRP	Age, BMI, education level, previous cesarean/miscarriage
Amiri et al. [28]	2013	Iran	Case-control	100/100	Carpenter and Coustan criteria	Second	Serum ferritin iron TIBC	Age, parity, body mass index, gestational age
Behboudi-Gandevani et al. [29]	2013	Iran	Prospective cohort	72/961	Carpenter and Coustan criteria	Second	Serum Iron, hemoglobin, total dietary iron	Age, BMI, education, job, parity, family history of GDM
Javadian et al. [30]	2014	Iran	Case-control	52/20	ADA,14	Second	Ferritin, hemoglobin	Gestational age, maternal age, weight
Helin et al. [31]	2012	Finland	Prospective cohort	68/321	Carpenter and Coustan criteria	First	Iron intake, hemoglobin	Maternal age and weight, total maternal weight gain, family history of diabetes, previous gestational diabetes or macrosomia, total calories intake, fiber, and saturated fatty acids intake

Discussion

Serum Ferritin and Risk of GDM

The goal of this study was to see if there was a link between serum ferritin and GDM. We discovered a positive correlation between high serum ferritin and GDM based on prospective cohort and case-control studies. High ferritin levels are thought to be linked to the development of GDM. Many studies have shown a greater incidence of diabetes in the general population in connection with increased iron stores [20,31,32]. Similar studies have found a significant association between high serum ferritin level and the risk of GDM in the pregnant population after adjusting prenatal BMI and other risk factors [24,29]. In a prospective case-control study on 1,384 patients by Soheilykhah et al., it was found that women with GDM had higher serum ferritin levels than the control. It was also concluded that a 45 ng/ml ferritin concentration was associated with a 1.4-fold increased risk of GDM [23]. Another study found that high ferritin levels of more than 80 ng/ml were associated with a 2.4-fold higher risk of GDM compared to those with low serum ferritin levels [28].

In another systematic review, the risk of GDM was significantly associated with high ferritin levels and heme iron [33]. Sharifi et al. identified high serum ferritin levels as an independent risk factor for GDM [34]. On the contrary, Zein et al. denied the association between high serum ferritin and GDM but found that excess iron plays a part in the development of impaired glucose tolerance [27].

Even though the biological mechanism involving high serum ferritin in the development of GDM is still unclear, Behboudi-Gandevani et al. identified it as a sensitive and specific predictor for diagnosing GDM [29,35]. Research has found that high serum or plasma ferritin reflects elevated iron stores of the body and may be considered an acute phase inflammatory reactant [22,36]. Pregnancy is a diabetogenic state. Furthermore, elevated serum ferritin levels trigger the inflammatory process causing reduced insulin secretion by the pancreas, increased insulin resistance, and hepatic dysfunction [37]. This ultimately resulted in decreased glucose uptake by muscles and increased gluconeogenesis, thus leading to the development of GDM [38]. Lao et al. have described high maternal hemoglobin as an independent risk factor for the development of GDM at 28-30 weeks [39].

Body Iron Stores and Risk of GDM

The individuals with high iron levels had three times greater risk of developing T2DM in the next 10 years after excluding the other risk factors like age, ethnicity, and BMI [32]. Maternal iron stores depend on pre-pregnancy iron stores, iron intake, and inflammatory status. At mid-trimester, serum ferritin concentration drops due to hemodilution and may reach to or equal to 50% of the early pregnancy level. To overcome this, more iron is mobilized from stores [40]. Guo et al. stated that elevated serum ferritin and iron level in early pregnancy have an association with the development of GDM [41]. Zein et al. showed in their study on Lebanese, non-anemic, non-supplemented pregnant women that the bioavailability of iron is greater in early pregnancy even if the woman has low iron intake. He also found a link between GDM and normal or modestly elevated iron reserves in the mid-trimester of pregnancy, indicating that iron supplementation should be tailored to the individual [27]. Researchers discovered that iron supplementation causes considerably increased plasma ferritin levels in pregnant women with normal hemoglobin levels, which are linked to the development of GDM [30].

sTfR and Risk of GDM

Soluble transferrin receptor (sTfR) is a marker for determining iron insufficiency at the tissue level. It has also been under consideration concerning GDM risk [26]. In the study by Rawal et al., the obtained sTfR and ferritin ratio was determined to be inversely associated with GDM risk, concluding that raised iron stores could be implicated in the development of GDM [25]. An acute inflammatory response may not affect the concentration of sTfR and may determine iron status in inflammatory conditions. sTfR and ferritin ratio may help assess iron store status and iron homeostasis at the cellular level [42,43]. One prospective and longitudinal study in the USA concluded a significant inverse relationship between GDM and the sTfR and ferritin ratio after adjusting a major risk factor for GDM; however, no association between GDM and sTfR concentrations was found [25]. The study by Soubasi et al. found that sTfR was not related to either high ferritin levels or excess iron stores, although, elevated maternal ferritin was found to be related to increased risk of GDM [42].

Hepcidin and Risk of GDM

Hepcidin is of key importance in the homeostasis of iron. Women with GDM displayed significantly elevated hepcidin levels. Hepcidin increases in response to elevated iron levels. It is associated with indices of glucose metabolism, such as fasting blood glucose, fasting insulin level, and glucose value response to the glucose challenge test [44]. A high hepcidin level may represent a risk of feto-maternal complication [45]. Rawal et al. discussed that in pregnancy, higher iron status (as represented by an elevated level of hepcidin and ferritin and a reduced ratio between sTfR and ferritin) was shown to be substantially associated with increased risk of GDM [25].

C-Reactive Protein and Risk of GDM

There is not a single iron biomarker available which indicates body iron status. The biomarkers studied are reliable when used in conjunction with each other [46]. C-reactive protein (CRP), the marker of inflammation, has also been associated with GDM [47]. However, presently, few studies are available to observe this correlation. Jiang et al. concluded an association of GDM with high CRP [19]. In addition, it was also found that high CRP is an individual risk factor for T2DM [48].

Iron Intake and Risk of GDM

Diabetes has been linked to dietary advanced glycation end-products, which are found significantly in processed red meats and high-fat animal products [11]. High dietary intakes of red meat and heme iron, mainly from animal sources, are linked with an increased risk of diabetes; however, no significant correlation was found between intake of non-heme iron, supplementary iron, total iron consumption, and the risk of T2DM [26]. Similarly, a study conducted on pregnant women concluded that consuming dietary heme in large quantities is linked with an elevated risk of GDM [9]. Another study described that high iron consumption enhanced the risk of GDM in pregnant women who had good hemoglobin levels greater than 12.0 g/dl in their early pregnancy [28].

Strengths and limitations

The strength of our research is that the selected studies are of high quality according to the JBI Critical Appraisal tool. We included the studies representing various races and ethnicities and studies conducted during the first and second trimesters of pregnancy. Our research highlights the importance of different iron biomarkers, whether conventional or novel.

Our study has a few limitations. Our search strategy was limited to studies in the English language and excluded the grey literature, leading to criteria bias. Secondly, the criteria used to diagnose GDM were not the same in all studies. The amount of oral glucose used was variable depending on the criteria used, and so different reference ranges for blood glucose levels to diagnose GDM. Another limitation was that our systematic review lacked third-trimester studies.

## Conclusions

High maternal serum ferritin levels play an important role in the development of GDM. We have found a relationship between high serum ferritin levels and the risk of GDM. High serum ferritin can be used as a diagnostic marker to assess GDM in pregnancy's first and second trimesters and also to assess body iron stores in pregnancy. High iron stores in the body are found to have a positive relation with GDM by producing oxidative stress, so assessing the ferritin level is, in other words, telling us about body iron stores. sTfR can be used to assess the iron level in any inflammatory condition instead of serum ferritin, but its role in the development of GDM is controversial. Serum hepcidin level can be used along with serum ferritin to assess the risk of GDM. We need more studies to measure the novel and conventional iron biomarker from preconception periods in women planning for pregnancy and women with a history of GDM in a previous pregnancy. The current findings are significantly important for clinical and public health to prevent the development of GDM in high-risk populations. It is also important to determine the appropriate routine supplementation and dose relationship in non-anemic and anemic pregnant women to avoid the development of GDM. This is especially important as certain populations meet the recommended dietary iron requirement and could be at risk of iron overload. As previous research has indicated a positive correlation between heme iron and T2DM, the role of heme iron in the development of GDM in pregnant women should be further investigated.
